# Shoot Organogenesis and Plant Regeneration from Leaf Explants of *Lysionotus serratus* D. Don

**DOI:** 10.1155/2013/280384

**Published:** 2013-08-01

**Authors:** Qiansheng Li, Min Deng, Jie Zhang, Wei Zhao, Yigang Song, Quanjian Li, Qingjun Huang

**Affiliations:** ^1^School of Ecology, Shanghai Institute of Technology, 100 Haiquan Road, Fengxian, Shanghai 201418, China; ^2^Shanghai Chenshan Plant Sciences Research Center, Chinese Academy of Sciences, Shanghai Chenshan Botanical Garden, Shanghai 201602, China; ^3^Genetic Diagnosis Center, Yunnan Provincial Key Laboratory for Birth Defects and Genetic Diseases, The First People's Hospital of Kunming, Yunnan Province 650032, China

## Abstract

The gesneriaceous perennial plant, *Lysionotus serratus*, has been used in traditional Chinese medicine. It also has a great development potential as an ornamental plant with its attractive foliage and beautiful flowers. An efficient propagation and regeneration system via direct shoot organogenesis from leaf explant was established in this study. High active cytokinin (6-benzyladenine (BA) or thidiazuron (TDZ)) was effective for direct organogenesis of initial induction. Murashige and Skoog (MS) growth media containing 0.5 mg L^−1^ BA alone or with combination of 0.1 mg L^−1^ **α**-Naphthaleneacetic acid (NAA) were the most effective for shoot proliferation. High BA concentration (1.0 mg L^−1^) in the media caused high percentage of vitrified shoots though they introduced high shoot proliferation rate. Histological observation indicated that adventitious shoot regeneration on the medium containing 0.5 mg L^−1^ BA alone occurred directly from leaf epidermal cells without callus formation. Regenerated shoots rooted well on medium containing half-strength MS medium with 0.5 mg L^−1^ indole-3-butyric acid (IBA) and indole-3-acetic acid (IAA), and the plantlets successfully acclimatized and grew vigorously in the greenhouse with a 94.2% and 92.1% survival rate.

## 1. Introduction

The plant family Gesneriaceae contains about 150 genera and 3700 species, which distributes worldwide primarily in tropical to temperate zones of eastern and southern Asia, Africa, Oceania, and South America to Mexico [1]. There are 58 genera and 463 species of this plant in China, among which 27 genera and 375 species are endemic, with more than half the species existing in narrow areas [2]. Many species of this family have very high ornamental value. The popular African violets (*Saintpaulia*), lipstick plant (*Aeschynanthus*), and gloxinias (*Sinningia*) are widely cultivated as houseplants. The appeal of gesneriads lies not only in their abundant, colorful flowers, but also in their attractive and colorful foliage. More and more people are starting to know and grow gesneriads as ornamental plants [3]. However, the overcollection for horticultural use and the destruction of natural habitat have caused many wild species of this family to become endangered. It is very necessary and urgent to cultivate and propagate them for horticultural use and biology conservation. Some species of the Gesneriaceae family in wide horticultural use have been propagated by tissue culture, such as *Saintpaulia* [4] and *Sinningia* [5, 6]. Even protoplast culture and fusion have been reported for horticultural breeding purposes [7, 8]. Other species of *Chirita *[9, 10]*, Metabriggsia ovalifolia *[11]*, Titanotrichum oldhamii *[12], and *Primulina tabacum* [13] were also propagated *in vitro* both for conservation and breeding purposes.

**Figure 1:**
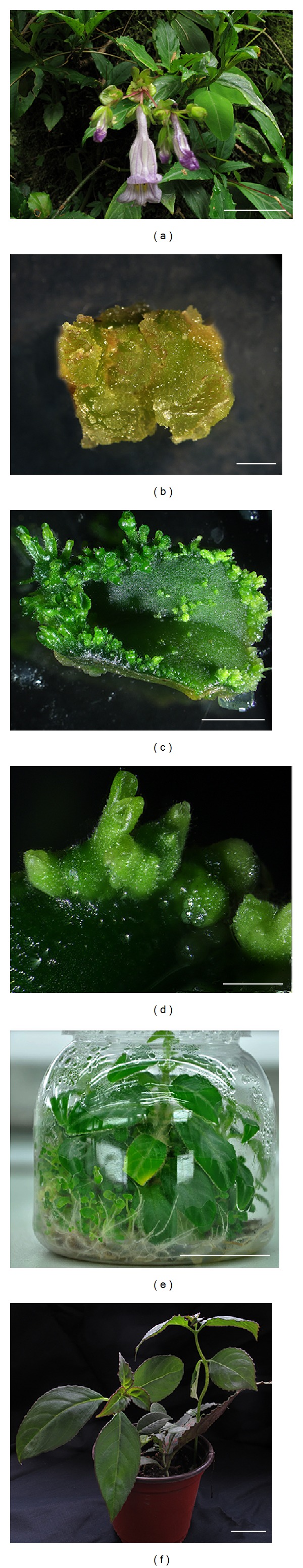
Morphogenesis of *Lysionotus serratus*. (a) Wild plant used for tissue culture, bar = 2 cm. (b) Calli appeared at the cut edge of a leaf explant on medium containing 0.5 mg L^−1^ KIN, bar = 2 mm. (c) Adventitious shoot induced from leaf explant culture on the induction medium containing 0.5 mg L^−1^ BA after 25 days, bar = 2 mm. (d) High magnification of induced adventitious shoots. bar = 1 mm. (e) Plantlet with well-established root system obtained by culturing leaf explant derived shoots on medium containing 0.5 mg L^−1^ IBA for 45 days, bar = 2 cm. (f) Regenerated plants 5 months after transplanting grew vigorously in a shaded greenhouse, bar = 2 cm.

*Lysionotus serratus* D. Don is a perennial herb under evergreen forests, with distribution ranging from western China to the eastern Himalayas [14]. This herb has attractive colorful leaves with silver veins and beautiful white to purple flowers, giving this plant high horticultural use potential as an indoor ornamental plant (Figure 1(a)). The species usually grows on trees or rocks in forests, streamsides, grassy slopes, or valleys. Due to increasing human disturbance in the forest systems, vast habitats of this herb have been destroyed. Several plants in this genus are harvested by local people as herbal medicine for treatment of lymph node tuberculosis, cough with tachypnoea, and rheumatic pains [15]. The functional compounds of their secondary metabolites have been isolated [16, 17]. Harvesting plants from the wild for medicine or horticulture purposes makes the species even more endangered. Therefore, it is urgent to establish an efficient and stable propagation method for conservation of this species and also for horticultural use. In this genus, *Lysionotus*, micropropagation has been reported on *Lysionotus pauciflorus* [18, 19]. However, no studies have been conducted on the cultivation, propagation, or breeding of this species for conservation and horticultural use. This study was conducted to establish an efficient *in vitro* plant regeneration system for *L. serratus* through leaf explants. It will be useful for mass production and also for future breeding by biotechnological methods such as protoplast fusion and genetic transformation.

## 2. Materials and Methods

### 2.1. Plant Material

Plants of *L. serratus *were collected from 1,850–2,000 m elevation, in Zhengyuan county, in the central part of Yunnan province, China. The plants were transplanted in plastic pots (Figure 1(a)) and grown for 6 months under a shaded greenhouse (maximum PPFD 550 *μ*mol m^−2^ s^−1^), where the day/night temperatures ranged from 15–32°C/10–25°C, depending on the season and local weather conditions.

### 2.2. Initial Induction and Shoot Organogenesis from Leaf Explants In Vivo

Healthy fully expanded young leaves, ranging from 30 to 40 mm in length, were harvested from potted mother plants and were used as initial explants. The leaves were soaked in 10x diluted liquid soap and rubbed gently by fingers prior to washing them under running tap water for 1 hour. The leaf surfaces were disinfected by 75% (v/v) ethanol for 10 s, followed by soaking for 10 min in 1 g L^−1^ HgCl_2_ solution and then rinsing 3 times with sterilized distilled water. The leaves were then cut into explants about 0.5 cm^2^ in size and inoculated onto MS basal media [20] supplemented with 0.5 mg L^−1^ BA, TDZ, KIN, or without any plant growth regulators (PGRs) for initial induction (Table 1). Each treatment contained 30 explants which were divided into 6 jars. The culture jars were 8.0 cm in height and 8.0 cm in diameter. All the media contained 30 g L^−1^ sucrose and 0.6% agar and were adjusted to pH 6.0 before autoclaving at 120°C and 105 kPa for 15 min. All culture jars were kept in an environmentally controlled growth room at 25 ± 2°C under a 12 h photoperiod with white fluorescent lamps (PPFD 40 *μ*mol m^−2^ s^−1^). After 4 weeks of culture, induction of shoot organogenesis was investigated and compared. Each treatment was replicated 3 times. 

After counting the numbers of adventitious buds of each explant, the clumps with adventitious buds were divided into smaller clumps with 3–5 buds and then transferred onto the same MS media with 0.5 mg L^−1^ BA to let shoots grow for another 4 weeks. These first generation *in vitro* shoots from the same media will be used as aseptic mother plants for PGR effects study.

### 2.3. Effects of PGR Concentration and Combination on Secondary Shoot Proliferation from Leaf Explants In Vitro

Leaves from the first generation *in vitro* shoots from the same media were then used as explants. They were cut into explants about 1.0 cm^2^ in area and inoculated onto MS basal media supplemented with different concentrations (0.1, 0.5, 1.0 mg L^−1^) of BA or TDZ, with or without the combination of 0.1 mg L^−1^ NAA (Table 2). The culture jars were cultured under a 12 h photoperiod at PPFD 40 *μ*mol m^−2^ s^−1^. Each treatment was done in 3 replications and each replication contained 50 explants in 5 jars. After 4 weeks' culture, induction of organogenesis was observed and the number of adventitious buds was counted. After that, the clumps with adventitious buds were divided into smaller clumps with 3–5 buds again and then transferred onto the same culture media for shoot formation and seedling growth for another 4 weeks. Each jar was transferred with 5 clumps. These second generation *in vitro* plantlets will be used as material for rooting. 

### 2.4. Plantlet Root Formation and Acclimatization

When the second generation adventitious shoots reached over 10 mm in height, they were transferred to half-strength MS media supplemented with 0.5 mg L^−1^ IBA, IAA, NAA, or PGRs free, respectively, for root formation. The shoots were incubated into culture jars that were 8 cm high and 8.0 cm in diameter; 4 weeks later, root formation was investigated. After a total of 30 days culture in the media, plantlets with 4–6 leaves and 3.0–5.0 cm in height were removed from the jars. The agar on the roots was gently removed by rinsing in tap water, and transplanted into a 6 cm plastic pot with sphagnum peat-based medium consisting of Canadian peat, vermiculite, and perlite in a 3 : 1 : 1 ratio based on volume. The growth medium was sprayed with water during mixing to achieve water content about 70%. Potted plantlets were grown in a low tunnel covered with plastic film on the bench of a shaded greenhouse with the maximum PPFD 200 *μ*mol m^−2^ s^−1^, temperature from 15 to 28°C, and relative humidity 75% to 90%. After transplanting, all the pots were watered again and sprayed with fungicide (1 mg L^−1^ carbendazim solution). The plastic film was kept closed to maintain the inside relative air humidity above 90% in the first 10 days, and then the plastic film was rolled up at night but kept partly closed during the daytime for another 5 days; after that the plastic film was fully removed. The survival rates of plantlets were recorded one month after transplanting.

### 2.5. Histological Investigation of Direct Shoot Organogenesis

Samples collected from different culture batches of leaf explants were taken weekly after culture initiation and were fixed in FAA (70% ethanol: formalin: acetic acid = 90 : 5 : 5 by volume). After dehydration through an ethanol-xylol series, the samples were embedded in a paraffin wax (58°C melting point). The sections, 8 *μ*m thick, were stained with Safranin/Fast Green and mounted on neutral balsam. All the sections were observed under a light microscope. 

### 2.6. Statistical Analysis

All the experiments of initial induction, shoot proliferation, and root formation were repeated 3 times. Data were analyzed by one-way ANOVA using the SPSS Statistics 19 and means were separated using Duncan's new multiple range test (*P* = 0.05).

## 3. Results

### 3.1. Initial Induction and Shoot Organogenesis from Leaf Explants In Vivo

The leaf explants showed no response to PGR-free medium in the first three weeks. Later, a few adventitious buds developed from the callus at the edge of cut surface (Table 1). 

On the medium containing 0.5 mg L^−1^ KIN, the leaf explants had no response for the first 3 weeks and then turned yellow and generally necrotic, without adventitious shoot formation (Figure 1(b)). 

The leaf explants showed no response to media containing 0.5 mg L^−1^ BA or 0.5 mg L^−1^ TDZ in the first two weeks. Thereafter, the leaf explants became swollen and some small shoot buds appeared directly on the leaf surface and cut surface in the explants placed on medium containing 0.5 mg L^−1^ BA, but no typical callus was formed (Figures 1(c) and 1(d)). On 0.5 mg L^−1^ TDZ supplemented medium, a mass of green compact calluses was formed after two weeks and adventitious buds appeared from both the calluses and leaf surface. The BA-containing medium induced more adventitious buds than medium containing TDZ (Table 1). Four weeks after these clumps of adventitious buds formed on the media containing 0.5 mg L^−1^ BA or TDZ, they were separated and transferred to the same media. Most of the buds developed well into shoots. 

### 3.2. Effects of PGR Concentration and Combination on Shoot Proliferation from Leaf Explants In Vitro

When the leaf explants of the first generation *in vitro* shoots from the same medium were cultured on the shoot proliferation media for a total of 4 weeks, the numbers of adventitious buds formation had significantly different responses to different concentrations of BA or TDZ, with or without the combination of NAA (Table 2). The highest number of buds (77.2) induced from leaf explants was found in the medium containing 1.0 mg L^−1^ BA only, but about half of them were vitrified after 4 weeks of culture, which indicated that the cytokinin concentration was too high. BA alone or any combination of BA with NAA induced more buds than TDZ with or without NAA at different concentrations, indicating that BA had stronger induction effects than TDZ. Considering the vitrification percentage, 0.5 mg L^−1^ BA with or without NAA was more effective for shoot proliferation. Somatic embryogenesis was not observed in the explants on any media in this study.

### 3.3. Root Formation and Seedling Acclimatization

Clumps of adventitious buds from the proliferation media were separated and transferred to the same media for further development of shoots. Four weeks later, the shoots were transferred to rooting media. The shoots developed quickly. They grew to 3.0–5.0 cm in height with their leaves developing 1.0–1.8 cm in length within 1 month. Adventitious shoots on the media containing auxins formed roots in 2 weeks, while the shoots on media without PGR formed roots 4 weeks after culture. The roots grew vigorously on media contain 0.5 mg L^−1^ IBA and IAA, respectively, with an average of 6–8 roots on each plantlet (Figure 1(e)); also, no calluses were induced on these two media. On medium containing 0.5 mg L^−1^ NAA, roots were fine, and calluses were formed at the cut end of adventitious shoots, but the induced roots turned brown after 4 weeks of culture.

After washing off the agar, 90 plantlets were separated from each rooting media and directly transplanted into 6 cm diameter plastic pots containing peat-based substrate. The highest survival rate (94.2%) was observed on the plantlets from medium containing 0.5 mg L^−1^ IBA, while the lowest survival rate (22%) occurred in PGR-free medium (Table 3). The surviving seedlings (Figure 1(f)) grew vigorously in the shaded greenhouses.

### 3.4. Histology of Direct Adventitious Shoot Formation from Leaf Explant

**Figure 2:**
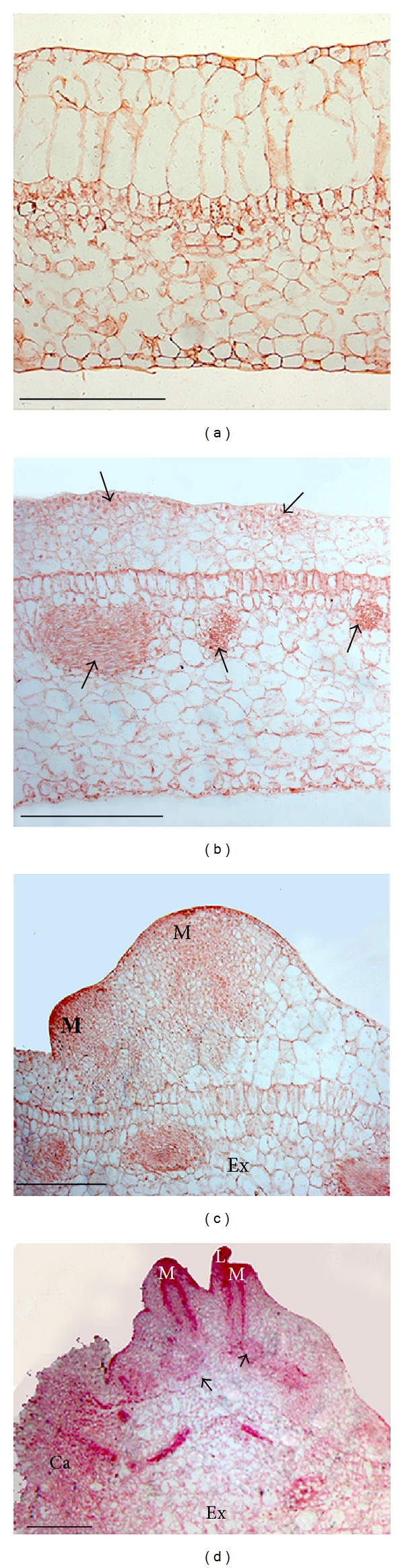
Histological observation of adventitious shoot formation from leaf explant of *L. serratus, *bar = 500 *μ*m. (a) Leaf explant before culture initiation. (b) Cell divisions in the adaxial leaf epidermis and close to the vascular bundles (*arrowheads*) 10 days after culture initiation on medium containing 0.5 mg L^−1^ BA. (c) Cell division to form adventitious shoot meristems (M) from explant (Ex) 20 days after culture initiation on medium containing 0.5 mg L^−1^ BA. (d) Adventitious shoot with leaf primordial (L) and shoot meristems (M) formed, with vascular bundles (*arrowheads*) connected to explant (Ex) 30 days after culture on medium containing 0.5 mg L^−1^ BA and 0.05 mg L^−1^ NAA. *Ca* indicates callus at the cutting edge.

Histological examination showed that fresh leaf explants had three layers of epidermis. The outer layer of the epidermal cells was small and impact. The other two layers of epidermal cells were prominent which were large and elongated. The palisade parenchyma was only one layer next to the epidermal cells, and the rest were well-developed spongy parenchyma (Figure 2(a)). Ten days after the initiation culture on medium containing 0.5 mg L^−1^ BA, cell divisions in the adaxial leaf epidermis and close to the vascular bundles (arrowheads) led to the formation of meristem cells (Figure 2(b)). But only the meristem formed by epidermal cells division developed to adventitious shoot meristems (M) from the explant (Ex) 20 days after culture initiation (Figure 2(c)). The adventitious shoot with leaf primordial (L) and shoot meristems (M) formed with vascular bundles (arrowheads) connected to the explant (Ex) 30 days after culture indicated that shoots were developed directly from the leaf epidermal cells (Figure 2(d)). Only a little callus formed at the cutting edges (indicated with Ca in Figure 2(d)), but no somatic embryogenesis was found.

## 4. Discussion

Because a large number of plants in the family Gesneriaceae are rosette-shape herbaceous, most tissue culture regeneration systems have been established from leaf blade explants in this family. Type and concentration of PGRs were critical factors influencing organogenesis and callus formation from leaf explants in members of the Gesneriaceae. Both shoot organogenesis or/and somatic embryogenesis were successfully induced in some species. For example, in *Chirita flavimaculata*, *C. eburnean, *and *C. speciosa, *adventitious shoots were induced from leaf explants without callus formation on medium with 0.1 mg L^−1^ of both NAA and BA [10]. In *Metabriggsia ovalifolia*, the highest shoot induction ratios were achieved by 2.5–5.0 *μ*M TDZ (or BA) and 0.25–0.5 *μ*M NAA [11]. In *Titanotrichum oldhamii*, adventitious shoots were formed efficiently on medium containing 0.1 mg L^−1^ BA [12]. In *Aeschynanthus radicans*, 40% of leaf explants produced somatic embryos on 6.81 *μ*M TDZ and 2.68 *μ*M 2,4-D medium [21]. In* Primulina tabacum*, somatic embryogenesis and shoot organogenesis could be switched simply by changing the order of TDZ and BAP (Benzylaminopurine) in the culture media [13]. These studies indicate that different species respond differently to PGRs, in terms of combination and concentration. High cytokinin level was essential to induce shoot organogenesis and somatic embryogenesis. However, plants in the Gesneriaceae seemed to be sensitive to BA, BAP, and TDZ, but not KIN. 

Different concentrations of TDZ and BA can lead to different morphogenesis response by explants, low concentration of TDZ has been shown to be beneficial for shoot organogenesis (e.g., *Saintpaulia*) [4], and high TDZ concentration can trigger somatic embryogenesis, in *Ochna,* for example [22, 23]. In the genus *Chirita*, when the medium contained high concentration of BA (>0.5 mg L^−1^) and lower NAA, adventitious shoots were easily induced, but when BA concentration was reduced to 0.1 mg L^−1^, both somatic embryos and adventitious shoots were induced [10].

In the present study, somatic embryogenesis was not induced in leaf explants of *L. serratus*, even with high concentration TDZ. The media containing 0.5 mg L^−1^ BA alone or 0.5 mg L^−1^ BA with 0.1 mg L^−1^ NAA resulted in a similar shoot induction ratio, but indirect shoots were found only on media containing NAA. Although NAA and IBA, as well as IAA, could all successfully induce the adventitious roots, NAA could prompt the formation of callus on the leaf explant. However, the roots induced by NAA were brown within 4 weeks with illness symptoms present on the plantlets, which indicated that NAA not only induced adventitious roots, but also prompted the accumulation of harmful secondary metabolites in the roots, which may directly inhibit the development of the roots and plant growth. On the contrary, IBA and IAA had no obvious differences on root induction. The roots grew vigorously and consistently in the media containing IBA and IAA. Therefore, both IAA and IBA were suitable for root induction for *L. serratus*. 

BA had better effects than the same concentration of TDZ on the induction of adventitious shoots in *L. serratus* leaf explants. Although high concentration BA could induce more shoots, about half of the shoots were vitrified which made them hard to develop into mature shoots or to induce roots on rooting medium. In conclusion, 0.5 mg L^−1^ BA concentration was ideal for adventitious shoot induction and 0.5 mg L^−1^ IBA or IAA was ideal for adventitious root induction for the rapid propagation of *L. serratus*. 

## Figures and Tables

**Table 1:** Effects of different cytokinins on shoot morphogenesis from leaf explants of *L. serratus. *

PGR (0.5 mg L^−1^)	Observation results on the leaf explants	Mean number of adventitious buds per leaf explant
0	Maintained green, few shoots	3.1 ± 0.2c^z^
0.5 BA	Little callus, adventitious shoots	36.4 ± 1.9a
0.5 TDZ	Compact callus, adventitious shoots	20.5 ± 1.2b
0.5 KIN	Little yellowish callus, no shoots	0.0 ± 0d

^z^Means with the different letters within a column indicate significant differences based on Duncan's new multiple range test (*P* = 0.05).

**Table 2:** Effects of PGRs on adventitious shoot proliferation in *L. serratus. *

PGR (mg L^−1^)	Adventitious bud number per explant	Morphology of the explants after 4 weeks of culture
0.1 BA	34.2 ± 1.8c^z^	Adventitious shoots
0.5 BA	52.6 ± 2.4b	Adventitious shoots with 2.5% shoots vitrified
1.0 BA	77.2 ± 4.8a	Adventitious shoots with 51.4% shoots vitrified
0.1 BA + 0.1 NAA	38.1 ± 2.2c	Adventitious shoots with compact callus
0.5 BA + 0.1 NAA	49.2 ± 2.5b	Adventitious shoots with compact callus, 5.5% shoots vitrified
1.0 BA + 0.1 NAA	66.4 ± 4.3ab	Adventitious shoots, with compact callus, 34.2% shoots vitrified
0.1 TDZ	22.1 ± 1.4d	Adventitious shoots, with few callus, shoot extremely small
0.5 TDZ	29.4 ± 1.8cd	Adventitious shoots, with few green compact callus
1.0 TDZ	18.4 ± 1.1d	Adventitious shoots, mass green compact callus, shoot extremely small
0.1 TDZ + 0.1 NAA	23.9 ± 2.1d	Adventitious shoots, mass green compact callus
0.5 TDZ + 0.1 NAA	25.4 ± 1.3d	Adventitious shoots, mass green compact callus, 8.5% shoots vitrified
1.0 TDZ + 0.1 NAA	33.5 ± 1.5c	Adventitious shoots, mass green compact callus, 19.6% shoots vitrified

^z^Means with the different letters within a column indicate significant differences based on Duncan's new multiple range test (*P* = 0.05).

**Table 3:** Influence of different auxins and activated charcoal on rooting and plantlet survival of *L. serratu*s.

PGR (mg L^−1^)	Culture time		Root formation in 4 weeks (%)	Survival rate of transplanted plants (%)
	2 wks	4 wks		
0.5 IBA	+	+	100	94.2a^z^
0.5 IAA	+	+	100	92.1a
0.5 NAA	+	+	100	68.1b
0	−	+	100	22.0c

“+” Indicates root formation, and “−” indicates that root was not formed within the observed time period. ^z^Means with the different letters within a column indicate significant differences based on Duncan's new multiple range test (*P* = 0.05).

## Abbreviations

BA: 6-Benzyladeneine
IAA: Indole-3-acetic acid
IBA: Indole-3-butyric acid
KIN: Kinetin
NAA: *α*-Naphthaleneacetic acid
TDZ: Thidiazuron.
